# Merkel cell polyomavirus and Langerhans cell neoplasm

**DOI:** 10.1186/s12964-018-0261-y

**Published:** 2018-08-22

**Authors:** Ichiro Murakami, Noriko Wada, Junko Nakashima, Mitsuko Iguchi, Makoto Toi, Yumiko Hashida, Tomonori Higuchi, Masanori Daibata, Michiko Matsushita, Takeshi Iwasaki, Satoshi Kuwamoto, Yasushi Horie, Keiko Nagata, Kazuhiko Hayashi, Takashi Oka, Tadashi Yoshino, Toshihiko Imamura, Akira Morimoto, Shinsaku Imashuku, Jean Gogusev, Francis Jaubert

**Affiliations:** 1 Department of Pathology, Kochi Medical School, Kochi University, Kohasu, Okoh, Nankoku, Kochi 783-8505 Japan; 2 Department of Pathology, Kochi University Hospital, 185-1 Kohasu, Okoh, Nankoku, Kochi 783-8505 Japan; 3 Department of Microbiology and Infection, Kochi Medical School, Kochi University, Kohasu, Okoh, Nankoku, Kochi 783-8505 Japan; 4 Department of Pathobiological Science and Technology, School of Health Science, Faculty of Medicine, Tottori University, 86 Nishi, Yonago, Tottori, 683-8503 Japan; 5 Department of Anatomic Pathology, Graduate School of Medical Sciences, Kyushu University, 3-1-1 Maidashi, Higashi-ku, Fukuoka, Fukuoka 812-8582 Japan; 6 Department of Pathology, Tottori University Hospital, 86 Nishi, Yonago, Tottori, 683-8503 Japan; 7 Division of Molecular Pathology, Faculty of Medicine, Tottori University, 86 Nishi, Yonago, Tottori, 683-8503 Japan; 8 Department of Virology, Okayama University Graduate School of Medicine, Dentistry and Pharmaceutical Sciences, 2-5-1 Shikata, Kita-ku, Okayama, Okayama 700-8558 Japan; 9 Department of Pathology, Okayama University Graduate School of Medicine, Dentistry and Pharmaceutical Sciences, 2-5-1 Shikata, Kita-ku, Okayama, Okayama 700-8558 Japan; 10 Department of Pediatrics, Kyoto Prefectural University of Medicine, Graduate School of Medical Science, 465 Kajii, Kawaramachi-Hirokoji, Kamigyo-ku, Kyoto, Kyoto 602-8566 Japan; 11 Department of Pediatrics, Jichi Medical University School of Medicine, 3311-1 Yakushiji, Shimotsuke, Tochigi, 329-0498 Japan; 12 Division of Laboratory Medicine, Uji-Tokushukai Medical Center, 145 Ishibashi, Makishima, Uji, Kyoto, 611-0041 Japan; 13 Inserm U507 and U1016, Institut Cochin, 75014 Paris, France; 14 AP-HP Hôpital Necker-Enfants Malades, University Paris Descartes (Paris 5), 75006 Paris, France

**Keywords:** Merkel cell polyomavirus, Langerhans cell neoplasm, Langerhans cell sarcoma, Langerhans cell histiocytosis, *BRAF* mutation, *RAS*/*MAPK* signaling pathway, Interleukin-1 loop model, Triple-factor model, ITIH4, Interleukin-17

## Abstract

**Background:**

The relationship between various external agents such as pollen, food, and infectious agents and human sensitivity exists and is variable depending upon individual’s health conditions. For example, we believe that the pathogenetic potential of the Merkel cell polyomavirus (MCPyV), the resident virus in skin, is variable and depends from the degree of individual’s reactivity. MCPyV as well as Epstein-Barr virus, which are normally connected with humans under the form of subclinical infection, are thought to be involved at various degrees in several neoplastic and inflammatory diseases. In this review, we cover two types of Langerhans cell neoplasms, the Langerhans cell sarcoma (LCS) and Langerhans cell histiocytosis (LCH), represented as either neoplastic or inflammatory diseases caused by MCPyV.

**Methods:**

We meta-analyzed both our previous analyses, composed of quantitative PCR for MCPyV-DNA, proteomics, immunohistochemistry which construct IL-17 endocrine model and interleukin-1 (IL-1) activation loop model, and other groups’ data.

**Results:**

We have shown that there were subgroups associated with the MCPyV as a causal agent in these two different neoplasms. Comparatively, LCS, distinct from the LCH, is a neoplastic lesion (or sarcoma) without presence of inflammatory granuloma frequently observed in the elderly. LCH is a proliferative disease of Langerhans-like abnormal cells which carry mutations of genes involved in the *RAS*/*MAPK* signaling pathway. We found that MCPyV may be involved in the development of LCH.

**Conclusion:**

We hypothesized that a subgroup of LCS developed according the same mechanism involved in Merkel cell carcinoma pathogenesis. We proposed LCH developed from an inflammatory process that was sustained due to gene mutations. We hypothesized that MCPyV infection triggered an IL-1 activation loop that lies beneath the pathogenesis of LCH and propose a new triple-factor model.

## Background

Langerhans cell neoplasms are divided into two distinct diseases, the Langerhans cell sarcoma (LCS) and Langerhans cell histiocytosis (LCH). Langerhans cells located in skin, function as sentinel or antigen-presenting cells that can capture invading viruses [1]. We discovered the relationship between Merkel cell polyomavirus (MCPyV) and these two diseases are similar to Epstein-Barr virus pathogenetic potential that by itself is involved in several neoplastic and inflammatory diseases (Table 1).

**Table 1:** Proposed relationship between viruses and cigarette smoking and host

Role	Cancer-causing	Inflammation-inducing
Epstein-Barr virus	Malignant lymphoma	Infectious mononucleosis
	Gastric cancer	Hemophagocytic syndrome
	Burkitt lymphoma	Necrotizing lymphadenitis
	Nasopharyngeal cancer	
Merkel cell polyomavirus	Merkel cell carcinoma	Langerhans cell histiocytosis (LCH)
	Langerhans cell sarcoma	
Cigarette smoking	Lung cancer, Pulmonary LCH	Chronic obstructive pulmonary disease, Pulmonary LCH

**Fig. 1:**
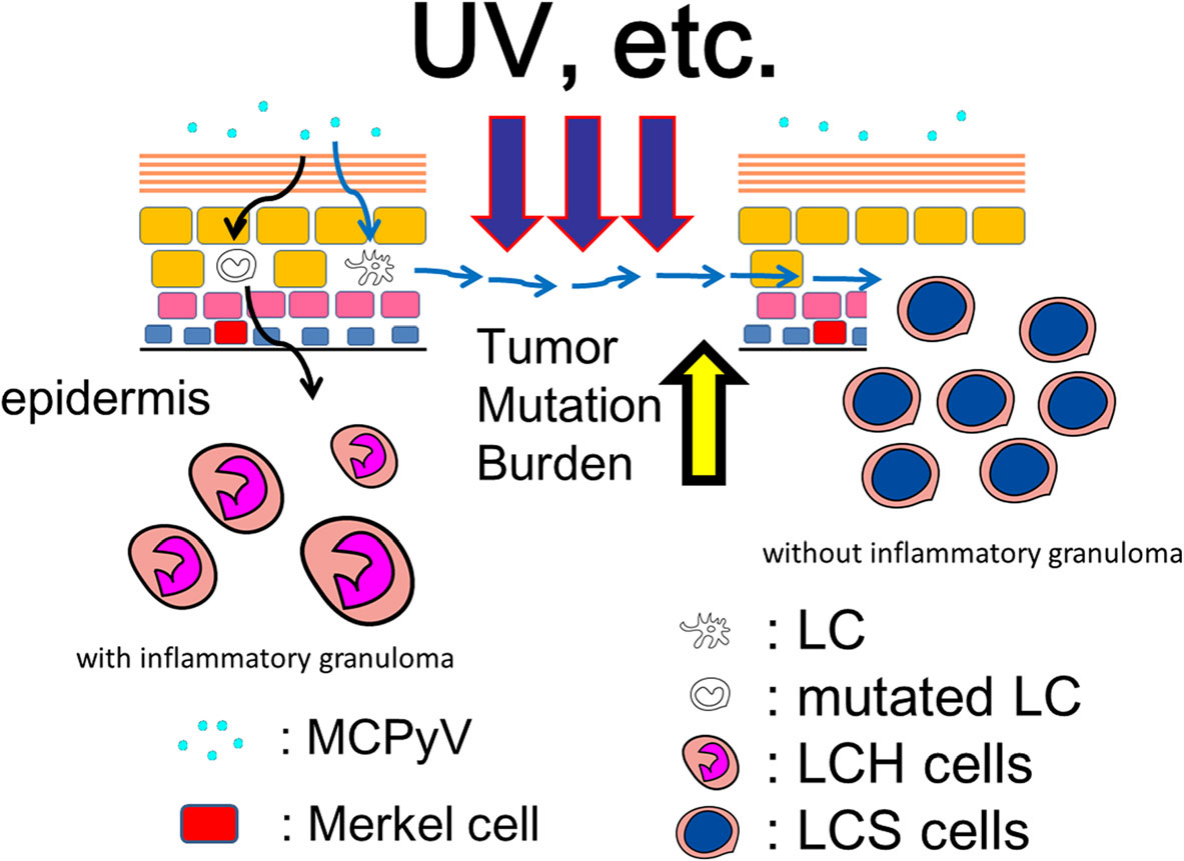
Proposed role of MCPyV in the development of LCH and LCS. We propose two distinct models for LCS and LCH pathogenesis. LCS is a malignant neoplasm initiated by MCPyV infection. On the contrary, LCH is a reactive disorder with underlying neoplastic potential. In other words, LCH is an inflammatory process that is prolonged by mutations. MCPyV: Merkel cell polyomavirus; LCH: Langerhans cell histiocytosis; LCS: Langerhans cell sarcoma; UV: ultraviolet; LC: Langerhans cell; LCH cells: CD1a-positive activated Langerhans (not atypical Langerhans cell, morphologically)-like cells in LCH lesion; LCS cells: sarcoma cells in LCS

In this review, we propose two distinct models for LCS and LCH pathogenesis (Fig. 1). Today, some LCS cases are considered as a malignant neoplasm initiated by MCPyV infection [2]. On the contrary, LCH is a reactive disorder with underlying neoplastic potential. In other words, LCH is an inflammatory process that is protracted by gene mutations, which we promote as an IL-1 loop model that was quoted in the WHO Classification of Tumours of Haematopoietic and Lymphoid tissues Revised 4th Edition in 2017 [2] as the major pathway in the development of Tumours derived from Langerhans cell.

We further propose a new triple-factor model for the pathogenesis of LCH.

### LCS

MCPyV was discovered in 2008 and was linked to the pathogenesis of Merkel cell carcinoma (MCC), which is a rare and aggressive skin cancer occurring in the dermis of individuals aged 60 years or older [3, 4]. Approximately 80% MCC harbors MCPyV, indicating its prominent role in the development of the disease. Mechanistically, MCPyV-induced oncogenesis is considered to be induced by MCPyV large T (LT) antigen through molecular binding with the retinoblastoma protein [3]. Several tumorigenic pathways leading to MCC were proposed. One was that the induced mutations of MCPyV due to long exposure to ultraviolet light leads to integration of the cytoplasmic viral sequences into the DNA of originating MCC cells. MCPyV might primarily reside in the skin, which was confirmed by the presence of MCPyV-DNA sequences of cutaneous tissue in our studies [5–7].

Langerhans cells are present beyond the middle of the spinous epidermal layer [8], they have the capacity to capture external pathogens [9], and can act as antigen-presenting cells [10, 11]. We previously proposed that external pathogens may be initially recognized by Langerhans cells and may subsequently infect Merkel cells which are mostly located at the basal cell layer of the epidermis (Fig. 1). Therefore, we hypothesized that Langerhans cells serve as a reservoir for MCPyV and demonstrated this phenomenon by showing the presence of MCPyV-DNA sequences in microdissected Langerhans cells from dermatopathic lymphadenopathy [12].

We hypothesized the possibilities that some LCS cases develop from a long standing reservoir cell for MCPyV and showed higher frequency of MCPyV-DNA sequences in LCS tissues with high viral load compared with that in non-affected normal Langerhans cells [2, 13].

### LCH

LCH is characterized by the proliferation of CD1a-positive activated Langerhans (not atypical Langerhans cell, morphologically)-like cells (LCH cells) generating inflammatory granuloma. LCH is classified by its involvement of either a single organ system (SS-LCH) or multiple organ systems (MS-LCH) [14]. The latter form is frequent in children younger than 2 years, whereas SS-LCH is more common in children older than 2 years [11, 15]. This rare disease affects 4–9 children per million each year [16–18]. The liver, spleen, and bone marrow (BM) are considered high-risk target organs for LCH [19, 20]. Therefore, LCH is also classified as involving at least one high-risk organ [LCH-RO (+)] or a no high-risk organ [LCH-RO (−)] [19] (Table 2) [21].

**Table 2:** Comparison between the present and former classification of LCH [21]

Classification			Prevalence
Present		Former	
LCH-RO (+)	MS	Letterer-Siwe disease	10%
LCH-RO (−)	MS	Hand–Schüller–Christian disease	20%
	SS	Eosinophilic granuloma	70%

LCH-RO (+): LCH involving at least one high-risk organ; LCH-RO (−) LCH involving a no high-risk organ; SS-LCH: LCH involving a single organ system; MS-LCH: LCH involving multiple organ systems

The morphology of lesions is so unvarying that pathologists cannot determine whether a given biopsy originates from a patient with SS-LCH or MS-LCH, from a patient with LCH-RO (+) or LCH-RO (−), or from a child or an adult [22]. However, the clinical course of LCH is remarkably variable, ranging from lesions that spontaneously resolve, to a chronic disease that can be widespread and sometimes lethal [23–26].

Although LCH was first described a century ago, the etiology is still not understood [27]. For decades, it was thought that the disease is a reactive disorder rather than a neoplastic process [27]. As the former name, “eosinophilic granuloma” indicates that lesional LCH morphology is reminiscent of tissue reactions to an intracellular pathogen, where tuberculous granuloma is the prototype [22]. For example, scabies infections are reported to induce Langerhans cell hyperplasia, which mimics LCH [28]. However, recent studies indicate that LCH has a more neoplastic character [29–31]. While unexpected remission can rarely occur in neoplasms, spontaneous healing is more common in LCH, suggesting that there may be multiple pathological factors contributing to the LCH process [22, 32, 33]. In this context, an epidemiologic study revealed that risk factors for MS-LCH involve an increase in infections, the use of antibiotics in the first 6 months of life, and a family history of thyroid disease, whereas SS-LCH is significantly associated with symptoms like diarrhea and vomiting in the postnatal period [34].

## Review

### LCS: High viral load of MCPyV-DNA

In previous studies we specified the relationship between MCPyV infection and LCS [2, 13]. Thus, we suggest that MCPyV may play specific role as an oncogenic factor in certain subtypes of LCS. Based on the foregoing, we propose an LCS tumorigenesis model where MCPyV may be a cause of LCS. In this regard, the recent discovery of MCPyV as a causal agent opened new therapeutic avenues for MCC [35]. Although MCPyV-LT expression was not detected [13], some forms of LCS might originate from MCPyV-infected Langerhans cell (Fig. 1). When confirmed, these findings will also open novel possibilities for therapeutic interventions against LCS.

### LCH: IL-17 endocrine model

Coury et al. found IL-17A to be elevated in the serum of patients with LCH and suggested that it might be involved in LCH pathogenesis according to the IL-17A autocrine model [36]. The IL-17A autocrine model in LCH and the IL-17A targeted therapies proposed by Coury et al. [36] have generated considerable controversy. Those authors showed high serum IL-17A levels in LCH and argued that serum IL-17A supported healthy monocyte-derived dendritic cell (DC) fusion capacities in vitro, rather than serum IL-17A levels, which is more correlated with LCH severity (i.e., the IL-17A paradox) [36]. On the contrary, Allen et al. [37–39] were unable to confirm the data presented in Coury et al. [36]. So started the controversy on the role of IL-17A [40, 41]. IL-17A is a proinflammatory cytokine produced by various cells including T helper type 17 cells (Th17), γδTcells, CD8+ T cells, natural killer T cells, lymphoid tissue inducer-like cells, neutrophils, monocytes, and natural killer cells [42–44]. IL-17A acts in both innate and acquired immunity [44]. Innate lymphoid populations can rapidly produce IL-17A [44], which is maintained at low levels in the absence of external stimulation [45]. Moreover, IL-17A/IL-17A receptor is highly important for host defense [46]. We approached the IL-17A controversy and the IL-17A paradox from a new perspective, i.e. considering the expression levels of IL-17A receptor, based upon what we propose an IL-17A endocrine model of LCH [47].

Our study about IL-17 [47] resulted in three major findings. First, the serum levels of IL-17A were higher in LCH as compared to controls with no significant differences among LCH subclasses. Second, higher levels of IL-17A receptor protein expression in MS-LCH were detected as compared to SS-LCH. Third, our results using LC/MS and LC/MRM-MS did not confirm the presence of IL-17A in LCH cells. An endocrine model supported our data: the IL-17A serum levels and expression levels of IL-17A receptor are higher in LCH tissue in patients with LCH. Accordingly, we postulate that the level of IL-17A receptor expression in LCH cells defines the LCH subclass [47]. We consider LCH as a reactive and neoplastic disorder that is influenced by environmental triggers such as pathogens or smoking. In this context, IL-17A is one of the proinflammatory cytokines acting against infective agents. A high serum IL-17A level might be considered to indicate the possibility of an infection in relation to LCH. Serum of patients with LCH can show an upregulation of IL-17A receptor in LCH cells but also in healthy monocyte-derived DCs. This hypothesis could elucidate the IL-17A paradox presented by Coury et al. [36]. In general, cytokines work throughout autocrine or paracrine mechanisms; however, IL-3 and some other proinflammatory cytokines exhibit endocrine mechanisms [47, 48]. In our analysis of LCH tissue, the results using LC/MS and LC/MRM-MS confirm the observation that the IL-17A reactivity in LCH cells observed by immunofluorescence [36] is due to nonspecific antibody binding as described by Allen et al. [37–39]. We resolved this problem by adding data of IL-17A RECEPTOR mRNA expression uploaded by Allen et al. [39]. Generally, stimuli are recognized by receptors such as Toll-like receptors (TLRs) in Langerhans cells [44, 49]. LCH cells also express these receptors in the GSE16395 dataset [39]. LCH cells that are in an active state [50] can induce IL-17A producers in a similar manner as activated Langerhans cells promote Th17 polarization [44]. In this context it would be useful to evaluate CD4/CD8 ratio and assess Th17 in peripheral blood of patients affected by LCH compared to healthy individuals. Though Allen et al. showed low serum levels of IL-17A [37, 38], Makras et al. showed high serum levels of IL-17A using the same enzyme-linked immunosorbent assay (ELISA) kit procedure in both: patients with LCH and controls without significant difference [41]. As IL-17A receptor is ubiquitously expressed [36, 44], it might be difficult to detect IL-17A in the blood as replied Delprat et al. to Allen et al. [37]. We analyzed patient’s sera using a Bio-Plex suspension array system (Bio-Rad), which is different from the other ELISA systems [37, 38, 41]. We found that the serum levels of IL-17A were higher in LCH as compared to controls with no significant differences among LCH subclasses. For host defense, IL-17A/IL-17A receptor complex is important [46], since IL-17A is commonly produced during viral infection [51]. In LCH, an overreaction by mutated LCH cells against stimuli such as viral agents might occur, including increased IL-17A receptor expression. In the context of infection, pathogens such as Epstein–Barr virus [52], human cytomegalovirus [53], and human herpes virus 6 [54, 55] were proven to exist in LCH cells. Although they were regarded as bystander in the LCH lesion in a case-controlled sero-epidemiological study and in situ analysis [11, 56], these investigations were done in 2008 before the discovery of *BRAF* gene mutations LCH cells in 2010 [31]. At present there is requirement to reexamine the health condition in patients with or without *BRAF* mutated precursor LCH cells. As reported using the LCH tissue [11, 57–60], serum levels of IL-1a and IL-6, which are known to stimulate Th17 [44], were also significantly higher as compared to controls. Our own analyses on LCH tissues using LC/MS and LC/MRM-MS could not confirm IL-17A positivity in LCH cells (i.e., the IL-17A autocrine model in LCH) [36]. Rather, we propose an IL-17A endocrine model and stress that alteratins in IL-17A receptor expression levels are important for defining LCH subclasses. Low IL-17A levels in sera are maintained by γδT cells in emergencies such as infection [45]. Allen et al. also showed that CD3-positive cells in tonsils produced IL-17A [37, 39]. In 2014, Lourda et al. investigated the presence of IL-17A-producing cells among peripheral blood mononuclear cells isolated from LCH patients and observed a high percentage of IL-17A(+) monocytes in peripheral blood of LCH patients compared to controls [61].

IL-17A/IL-17A receptor signaling pathways include matrix metalloproteinase-3 (MMP3) or MMP12 [62–64]. These MMP3 and MMP12 belong to a series of 1410 genes, the levels of which were more than twofold higher in LCH cells as compared to Langerhans cells in the re-analysis of GSE16395 mRNA data. These higher expression levels of MMP3 and MMP12 not only confirm IL-17A/IL-17A receptor signaling roles in LCH cells but also explain the inflammatory process of LCH such as bone absorption and accumulation of eosinophils [65–67]. In summary, LCH is a neoplastic disorder driven by abnormalities such as *BRAF* gene mutation [31] thus the severity of LCH might be driven by an inflammatory process under the form of a cytokine storm, especially involving IL-17A/IL-17A receptor signaling pathways. In the future, stimuli that govern IL-17A or IL-17A receptor production might serve as therapeutic targets to stop LCH progression, similar to cessation of smoking which induces pulmonary LCH regression [11, 68], which is almost always a disease of smokers [2].

### LCH: IL-1 loop model

Patients with LCH often have dermal disorders such as seborrheic dermatitis [19] concomitant to LCH lesions [69], preceding [70–72], or following LCH lesions [73]. We recently described the possibility of a causal relationship between LCH and dermotropic MCPyV [12], which was discovered as the major pathogenic agent in MCC of the skin in 2008 [3]. Our data indicate that MCPyV-DNA sequences are present in LCH tissues excluding pulmonary LCH, with significant differences between LCH tissues and controls that included patients with dermatopathic lymphadenopathy and reactive lymphoid hyperplasia [12]. The numbers of MCPyV-DNA sequences in LCH tissues from patients younger than 2 years indicated a significant difference from tissues of non-LCH dermal disease patients of the same age [12]. Our data suggest that LCH is a reactive disorder with an underlying oncogenic potential. Thus, both LCH and pulmonary LCH harbor the *BRAF* V600E mutation [31, 74, 75] and *NRAS* mutation [76] and appear related to external stimuli such as viral infection [12, 77, 78] and cigarette smoking [79, 80]. In addition, the removal of such stimuli is reported to cause spontaneous healing of LCH [68, 81–83].

Expression of the constitutively active *BRAF* V600E mutant in LCH cells is predicted to bypass the requirement for mitogen-induced activation of RAF by RAS [31, 84]. The identification of activating *BRAF* mutations supports the hypothesis that LCH is a process with oncogenic potential [31]. A mouse LCH model using a *BRAF* V600E construct under the control of CD11c promoter and a *BRAF* V600E construct under control of the langerin promoter indicates that the *BRAF* V600E is not only a marker but also an essential driver of LCH pathogenesis [85]. Moreover, phosphorylated extracellular signal-regulated kinase (ERK) (pERK) is rapidly dephosphorylated by dual specificity phosphatase 6 (DUSP6) [86, 87], which is overexpressed in LCH cells [39]. However, *BRAF* V600E gene mutations are also detected in non-neoplastic disorders such as nevus cell nevus [88] and hyperplastic polyps of the colon [89]. Thus, LCH pathogenesis requires both limited proliferation of precursor LCH cells harboring the *BRAF* V600E mutation and the accumulation of gene mutations or an inflammatory trigger that activates the RAS/RAF/MEK/ERK signaling pathway [84].

MCPyV interferes with the function of DC towards evasion of the immune surveillance by targeting a NF-κB essential modulator [90] and down-regulating TLR9 [91]. Exposure to MCPyV as measured by serum antibodies against the viral capsid proteins appears to be widely prevalent among healthy subjects [92, 93]. Inapparent existence of MCPyV is indicated on the skin and environmental surface [94, 95]. Pancaldi et al. [96] indicated that buffy coats of healthy adult blood donors, which were examined for MCPyV-DNA tag sequences, showed a prevalence of 22%, with viral loads ranging from 10 to 100 molecules per 100,000 cells (0.0001 to 0.001 per cell). Mertz et al. [97] reported that CD14+ CD16− inflammatory monocytes are a reservoir for MCPyV, but CD14^low^CD16+ resident monocytes, lymphocytes, or granulocytes are not. Our data from micro-dissected LC in both dermatopathic lymphadenopathy [12] and LCS [13] suggest that monocytes, precursor Langerhans cells, or Langerhans cells are one of the reservoir cells for MCPyV. In addition, members of the TLR/IL-1 receptor superfamily appear to play a fundamental role in the immune response [98]. Viral “pathogen-associated molecular patterns” are recognized by specific TLRs [99]. TLR agonists stimulate IL-1β production in DC [100], where TLR-triggered ERK activation play important roles [101]. IL-1α expression is induced by TLR-mediated NF-κB activation; such activation has been observed in some LCH cases [102, 103], with/without the presence of IL-1β [104]. All TLRs except TLR3 use the common MyD88-dependent pathway [105]. MyD88 is one of the adaptor proteins that links TLR/ IL-1 receptor [106] and binds to pERK via its D-domain, thereby preventing pERK-DUSP6 interaction and maintaining ERK in an active, phosphorylated state for a longer period [86]. This MyD88-dependent signal may lead to enhanced cell activation, proliferation, and eventually, accumulation and prolonged survival [86, 107] of a given LCH lesion [108].

### LCH: ITIH-4

Interalpha-trypsin inhibitor heavy chain 4 (ITIH4, [PDB: Q14624]) is an acute-phase-related protein [109] and potential new biomarker for distinguishing MS-LCH and SS-LCH. Acute-phase proteins are involved in non-specific, physiological immune functions within the innate immune system [110]. The ITIH4 molecule has been detected in animals during experimental bacterial and viral infections [111].

Martel-Jantin et al. [112] reported seroprevalence rate of MCPyV antibodies of children 12 months or younger (49/105) in Cameroon and pointed out the presence of specific maternal antibodies in very young children. Their data indicated that MCPyV infections mostly occurred during early childhood, after the disappearance of specific maternal antibodies [112]. On the contrary Tolstov et al. [93] reported seroprevalence rate of MCPyV antibodies of children of 1 year or younger (0/6) in patients with LCH. We [12] identified a relationship between LCH and MCPyV. MCPyV-DNA in PBMC correlated with LCH-RO (+) [12]. Among patients with LCH-RO (−) (MS-LCH and SS-LCH), MCPyV-DNA was restricted to lesional LCH cells [12], thus we predicted that primary MCPyV infection may influence the LCH subtype involving cells in an early-activated state [27].

Generally, no response is observed after secondary viral infection [111]. For example, primary respiratory syncytial virus infection at 6 months or earlier often induces severe disease [113], although nearly all children are infected by 2–3 years of age [114]. Similarly, primary Epstein-Barr virus and cytomegalovirus infections in elderly individuals cause a severe condition called infectious mononucleosis; nonetheless, nearly all children are infected with these viruses [115]. Although no response is observed after MCPyV infection [94, 96], Kumar et al. [116], however, found that MCPyV-specific T helper cells (in vitro model of a secondary infection) secrete several cytokines, including IL-10. IL-10 is an anti-inflammatory cytokine and is one of cytokines to be produced in LCH [21]. ITIH4 production is up-regulated by IL-6 [109], which is known produced in LCH [21]. Innate immune function between newborns and elderly is extremely different and large quantities of IL-6 after stimulation of receptors, such as TLR, by term newborns are indicated [117]. In LCH, MCPyV infection may induce hyper-immunity in both LCH cells [108] and other inflammatory cells [11, 21].

### LCH-RO (+) and LCH-RO (−)

We reported the presence of MCPyV-DNA in the peripheral blood cells of patients with LCH-RO (+) but not in the blood cells of patients with LCH-RO (−) [12]. Berres et al. [85] reported that patients with LCH-RO (+) carried the *BRAF* V600E mutation in circulating CD11c+ and CD14+ cellular fractions as well as in bone marrow CD34+ hematopoietic cell progenitors, whereas the mutation was restricted to lesional LCH cells in patients with LCH-RO (−). These findings (Table 3) specifically observed in LCH-RO (+) suggest the LCH pathogenetic pathway, though it needs further confirmation to conclude.

**Table 3:** Detection of MCPyV-DNA and *BRAF* mutation in PBMC of patients with LCH

Classification	MCPyV-DNA	*BRAF* mutation
LCH-RO (+)	(+)	(+)
LCH-RO (−)	(−)	(−)

Status of PBMC (peripheral blood mononuclear cells) of patients with LCH based on both our and other researcher’s data [12, 85]

### Pulmonary LCH

The incidence of *BRAF* mutation did not differ significantly [31] between pulmonary LCH that has been regarded as reactive to smoking [11, 74, 79] and non-pulmonary LCH that has been regarded as a neoplastic process [11, 29–31, 118]. Since smoking increases the number of Langerhans cells in chronic obstructive pulmonary disease [119], precursor LCH cells may overreact to smoking. Similarly in cutaneous LCH, overreaction to stimuli such as a dermotropic MCPyV infection may occur [12].

### Spontaneous regression in LCH: Triple-factor model

Recently, congenital “self-healing” LCH (Hashimoto-Pritzker disease) condition was proposed as a model of LCH where Kansal et al. identified V600D mutation in Exon 15 of the *BRAF* gene [120]. As shown in Table 2 [21], patients with SS-LCH account for the majority of LCH patients. While unexpected remission can rarely occur in neoplasms, spontaneous healing is more common in LCH especially in SS-LCH, suggesting that there may be multiple pathogenetic influences to the LCH process [22, 32, 33].

In pulmonary LCH, the removal of stimuli, i.e. cessation of smoking, is well known cessation process for spontaneous healing [68, 81–83]. However, recent data indicate both LCH and pulmonary LCH harbor the *BRAF* V600E [31, 74, 75] and *NRAS* mutation [76] and appear linked to external stimuli such as viral infection [12, 77, 78] and cigarette smoking [79, 80].

We think that spontaneous healing in both LCH and pulmonary LCH suggest an oncogene-induced senescence [121] according to Chilosi, et al. who considered that oncogene-induced senescence distinguishes indolent from aggressive forms of pulmonary LCH and non-pulmonary LCH [122].

Using an in vitro model, Lipsky et al. [107] demonstrated that IL-1 production and signaling from the IL-1 receptor are necessary components of Raf-induced transformation of NIH 3 T3 cells, which exclude other factors involvement in the vivo model [85].

**Fig. 2:**
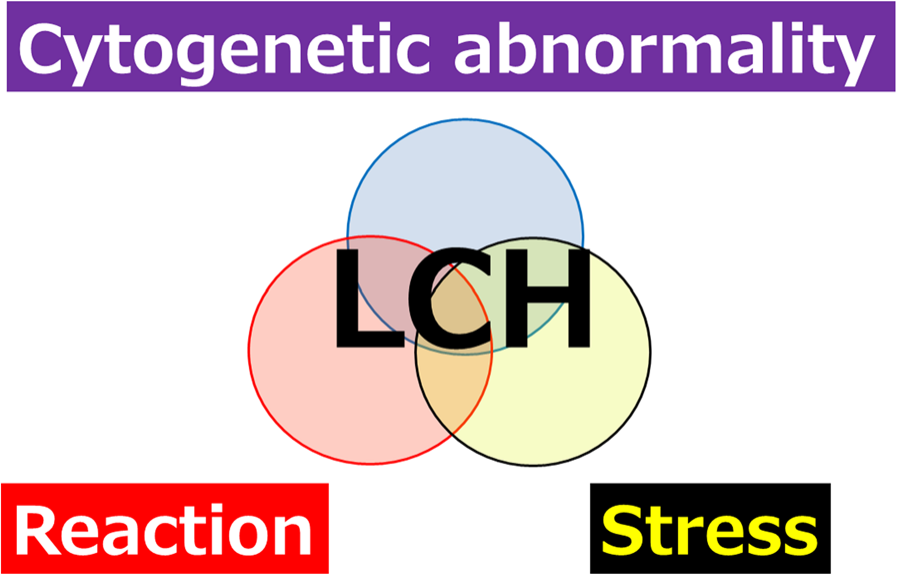
Proposed triple-factor model for LCH pathogenesis. We propose a triple-risk factor model for LCH pathogenesis. Triple-risk factor model is composed of three factors: cytogenetic abnormalities such as *BRAF* mutation, stress such as MCPyV infection and cigarette smoking, and reaction

**Fig. 3:**
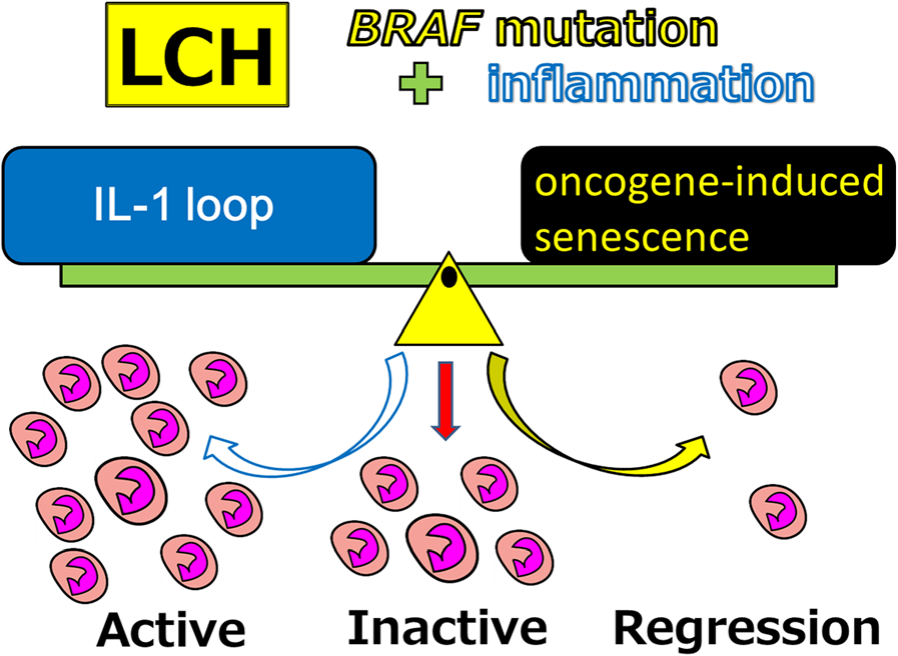
Proposed reaction model in LCH activity. We propose a triple-risk factor model for LCH pathogenesis. Triple-risk factor model is composed of three factors: cytogenetic abnormalities such as *BRAF* mutation, stress such as MCPyV infection and cigarette smoking, and reaction controlled by balance between an oncogene-induced senescence and an IL-1 loop. An oncogene-induced senescence is induced by cytogenetic abnormalities such as *BRAF* mutation and *MEK* mutation. An IL-1 loop is triggered by stress such as MCPyV infection, EBV infection, and cigarette smoking. An IL-1 loop may induce cytokine storm

The only *BRAF* V600E mutation does not seem to affect prognosis [2, 123]. We proposed a triple-factor model for pathogenesis of LCH (Fig. 2). We think that balance between oncogene-induced senescence [121] and the requirement of IL-1 autocrine loop [2, 107, 108] of *BRAF* V600E mutation in pulmonary and non-pulmonary LCH indicates the clinical severity of the disease (Fig. 3).

### Bone lesion in LCH

Approximately 80% of patients affected by LCH indicate presence of lesions in bone [124]. There are also bone lesions in recurrent LCH, even if it develops as MS-LCH, knowing that recurrent lesions may occur only in bone. In the LCH lesions, multinucleated giant cells coincide in bone as well as in skin and lymph nodes. In such conditions, tartrate-resistant acid phosphatase (osteoclast marker), vitronectin receptor, cathepsin K, and MMP9 are readily detected [125]. Bone homeostasis is a complex process controlled not only by bone metabolic cells but also by interaction with other distant tissues and cells. Both bone and immune system share many specific proteins such as cytokines that form a functional network [126, 127]. In this regards, Cathepsin K protease is important for bone resorption by osteoclasts, compare to DCs, where it acts to regulate signals from TLR9 and is involved in the Th17 cells differentiation [128]. Many immune system cytokines are involved in bone metabolism, such as involvement of IL-1, IL-6 in the heightening of bone resorption by osteoclasts also producing a cytokine storm. In particular, the receptor activator of nuclear factor kappa-B ligand (RANKL) is regarded as the most important protein [129]. RANKL is expressed on T cells and osteocytes [130] and is an essential protein in activation of DCs and differentiation of osteoclasts. In addition, the serum sRANKL value is significantly higher in LCH patients than in the control group [131]. We have found that MCPyV-DNA is also amplified in bone LCH [12]. MCPyV not only exists in the skin [5, 6, 94] but also exists in the blood of healthy people [96] and MCPyV is recognized by precursor LCH cells and appears to induce LCH lesion formation in bone which has so-called cytokine storm in order to keep homeostatic microenvironment. On the other hand, it has been pointed out that RANKL produced by regulatory T cells is involved in the metastasis of breast cancer [132]. In LCH, it is known that many regulatory T cells exist in lesions [23] and play a similar role in LCH bone lesion formation. Studies and treatments focusing on these relationships have also been realized [131, 133, 134].

## Conclusions

We have proposed that there are subgroups of clinical conditions associated with MCPyV designated as two different Langerhans cell neoplasms, LCS and LCH in comparison to Epstein-Barr virus and cigarette smoking pathogens (Table 1, Fig. 1).

Although there is a case report indicating progression from LCH to LCS [135] and case reports of detection of *BRAF* mutation in LCS [136, 137], these two diseases are not in the same spectrum considering age distribution, neoplastic cell morphology, inflammatory granuloma, and cytokine storm release.

**Fig. 4:**
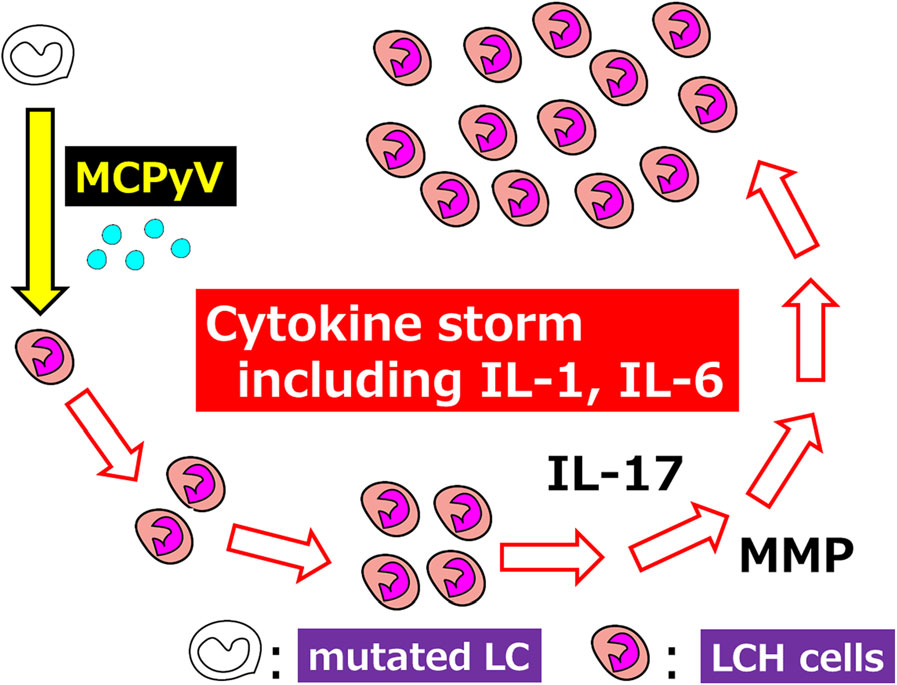
Proposed role of MCPyV in the development of LCH. We propose LCH is a reactive disorder with underlying neoplastic potential using a triple-risk factor model. Infection usually triggers cytokine production and antibody production. However, MCPyV infection triggers cytokine storm including IL-1 and IL-6 in LCH patients. IL-1 and IL-6 stimulate IL-17 producing cells. We found IL-17A receptor expression levels in LCH cells are important for defining LCH subclasses. IL-17/IL-17 receptor signaling pathways include matrix metalloproteinase-3 (MMP3) or MMP12 which induce bone absorption. MCPyV: Merkel cell polyomavirus; LCH: Langerhans cell histiocytosis; LC: Langerhans cell; LCH cells: CD1a-positive activated Langerhans (not atypical Langerhans cell, morphologically)-like cells in LCH lesion

Finally, there is a correlation between LCH subtypes and inflammatory factors such as expression of ITIH4 molecule [138] and IL-17A receptor [47]. However, there are no specific pathological definitions between the LCH subtypes such as self-healing SS-LCH, LCH-RO (−), and life-threatening LCH-RO (+). The principal pathological characteristics of LCH include the morphologic aspects of activated Langerhans cells adjoined to inflammatory granuloma. Therefore, we propose that LCH entity is an inflammatory process that is protracted by gene mutations occurring in the LCH cells interacting with other immunologically competent cells (Fig. 4) [108].

## Abbreviations

DC: Dendritic cell
DUSP6: Dual specificity phosphatase 6
ELISA: Enzyme-linked immunosorbent assay
ERK: Extracellular signal-regulated kinase
ITIH4: Interalpha-trypsin inhibitor heavy chain 4
LCH cells: CD1a-positive activated Langerhans (not atypical Langerhans cell, morphologically)-like cells
LCH: Langerhans cell histiocytosis
LCS: Langerhans cell sarcoma
LT: large T
MCC: Merkel cell carcinoma
MCPyV: Merkel cell polyomavirus
MMP: Matrix metalloproteinase
RANKL: Receptor activator of nuclear factor kappa-B ligand
Th17: T helper type 17 cells
TLR: Toll-like receptor
